# Sleep characteristics and hearing loss in middle-aged and older adults: The National Health and Nutrition Examination Survey 2015–2018^✰^

**DOI:** 10.1016/j.sleepe.2024.100082

**Published:** 2024-04-28

**Authors:** Kening Jiang, Adam P. Spira, Nicholas S. Reed, Frank R. Lin, Jennifer A. Deal

**Affiliations:** a Cochlear Center for Hearing and Public Health, Johns Hopkins Bloomberg School of Public Health, Baltimore, MD, USA; b Department of Epidemiology, Johns Hopkins Bloomberg School of Public Health, Baltimore, MD, USA; c Department of Mental Health, Johns Hopkins Bloomberg School of Public Health, Baltimore, MD, USA; d Department of Psychiatry and Behavioral Sciences, Johns Hopkins School of Medicine, Baltimore, MD, USA; e Johns Hopkins Center on Aging and Health, Baltimore, MD, USA; f Department of Otolaryngology-Head and Neck Surgery, Johns Hopkins School of Medicine, Baltimore, MD, USA

**Keywords:** Aging, Hearing loss, Sleep duration, Daytime sleepiness, Sleep-disordered breathing

## Abstract

**Background:**

Population-based evidence linking sleep characteristics with hearing is limited and how the associations change with age remains unknown. We aim to investigate cross-sectional associations between sleep characteristics and hearing by age in a nationally representative sample of U.S. adults.

**Methods:**

We included 3,100 participants aged 40 years and older from the National Health and Nutrition Examination Survey 2015–18. Audiometric hearing thresholds at 0.5, 1, 2 and 4 kilohertz were averaged to calculate better-ear pure-tone average (PTA). Sleep questions were self-reported, including sleep duration on weekdays or workdays, snoring, snorting or stopping breathing, trouble sleeping, and daytime sleepiness. Multivariable-adjusted linear regression with an interaction term between sleep characteristic (categorical) and age (continuous in years) was used. Primary models adjusted for demographic and lifestyle factors with additional adjustment for cardiovascular factors in secondary models.

**Results:**

There was no association of reported sleep duration with PTA at age 50; however, compared to those reporting 7–8 h of sleep, there was a borderline-significant association at age 60 (<7 h: 1.72 dB, 95 % confidence interval [CI], −0.21, 3.66; >8 h: 1.25 dB, 95 % CI, −0.01, 2.51), and a significant association at age 70 (<7 h: 2.52 dB, 95 % CI, −0.27, 5.31; >8 h: 2.67 dB, 95 % CI, 0.56, 4.79). No consistent associations for other sleep characteristics were found.

**Conclusions:**

Long sleep duration is associated with worse hearing among middle-aged and older adults and the association differs by age. Longitudinal evidence is needed to establish temporality and examine changes in hearing associated with sleep characteristics.

## 1. Introduction

Approximately 40 million US adults have hearing loss and the burden of hearing loss is concentrated among older adults [1]. The prevalence of hearing loss increases with age, and it is estimated to increase from 7 % in the forties to over 50 % in the seventies [1]. As the fourth leading cause of years living with disability [2], hearing loss has been associated with poorer health outcomes including cognitive decline and dementia [3,4], impaired physical function [5], social isolation [6] and depression [7].

These far-reaching consequences necessitate public health approaches for hearing loss prevention [2,8]. Identifying and characterizing risk factors for hearing loss is a key step towards this goal. In addition to demographic factors including age, sex and race that are strongly associated with hearing loss, sleep disturbances might be novel risk factors that are of public health significance [9]. Sleep disturbances, including short and long sleep duration, sleep-disordered breathing (SDB), insomnia symptoms (e.g., difficulty falling or staying asleep), and excessive daytime sleepiness are prevalent among older adults and are modifiable [10,11]. Sleep disturbances potentially contribute to hearing loss through decreased oxygen supply to the cochlea due to hypoxia from SDB or cardiovascular factors more broadly [12]. Other mechanisms, including inflammation and neurological changes have also been proposed [13,14].

Recent longitudinal evidence provided preliminary support linking sleep quantity and quality with subsequent hearing loss [15,16]. Several population-based studies in the United States and other countries have reported cross-sectional associations between sleep disturbances, especially sleep duration, with hearing loss [17–22]. However, prior studies primarily focus on links between SDB and hearing loss and existing evidence mainly comes from occupational cohorts and clinical samples, limiting the generalizability of findings [23–29]. Further population-based epidemiologic evidence, especially in nationally representative samples, is needed to better understand the sleep-hearing association and how the association might differ by demographics.

Our previous work on sleep and hearing among adults aged 70 years and older reported worse hearing among participants with long sleep duration in the National Health and Nutrition Examination Survey (NHANES) 2005–06 [17]. This study aims to build upon our previous NHANES study to examine the cross-sectional associations of sleep characteristics with audiometric hearing among a nationally representative sample of middle-aged and older adults using data from NHANES 2015–18. We hypothesize that sleep disturbances are associated with hearing loss. With higher prevalence of both sleep disturbances and hearing loss with older age, we also explored how the associations might differ by age.

## 2. Material and methods

### 2.1. Study population

The National Health and Nutrition Examination Survey (NHANES) is an ongoing biannual survey assessing health status of the noninstitutionalized civilian U.S. population. The NHANES sampling procedure uses a complex and four-stage (Stage 1: primary sampling units; Stage 2: segments; Stage 3: dwelling units or households; Stage 4: individuals) design. Each participant was assigned a sampling weight to obtain nationally representative estimates, accounting for unequal probabilities of selection, non-response, and adjustments to match the noninstitutionalized civilian U.S. population totals. Detailed sample design and estimation procedures have been published previously [30]. The National Center for Health Statistics Ethics Review Board approved all NHANES protocols with written informed consent obtained from all participants.

We used data from 2015–16 (*N* = 9971) and 2017–18 (*N* = 9254) cycles of the NHANES. Audiometry examinations were administered to a subsample (2015–16: aged 20–69 years; 2017–18: aged 6–19 and 70+ years) of NHANES participants examined in the mobile examination center (MEC). We limited our study population to 7648 participants aged 40 years and older, considering different etiologies of hearing loss among adolescents and young adults, and 3679 of them participated in the audiometry exam. We further excluded 275 participants missing audiometric data, 37 participants missing sleep questions, and 267 participants missing covariates, leaving a final study population of 3100 participants. We used separate analytic samples for two of the sleep questions - frequencies of snoring (*N* = 2841) and snorting or stopping breathing (*N* = 2873) due to relatively high missingness of these two questions.

Among 3679 middle-aged and older participants with audiometry, compared to the 3100 participants in our study population, the 579 participants with missing data were older, less likely to self-identify as non-Hispanic White, had lower educational attainment and lower body mass index (Table S1).

### 2.2. Hearing assessment

Audiometry examinations were conducted by trained personnel in a sound-isolating booth in MEC using Interacoustics AD226 audiometer (Interacoustics A/S, Assens, Denmark) or Audiometric Research Tool (ART) system with TDH-49P standard headphones and ER-3A insert earphones. Detailed audiometry procedures have been published [31, 32]. Audiometric hearing thresholds in decibels (dB) at 0.5, 1, 2 and 4 kilohertz (kHz) were obtained for both ears and were averaged to calculate 4-frequency pure-tone average (PTA). Better-ear PTA was analyzed both continuously and categorically as having hearing loss (>25 dB) or normal hearing (≤25 dB) according to clinical cut-off points [33].

### 2.3. Sleep assessment

Participants aged 16 years and older responded to questions on sleep habits and disorders as a part of the in-home interview. Sleep duration, as our primary exposure of interest, was calculated from usual time in and out of bed for sleeping on weekdays or workdays self-reported by participants and were rounded to the nearest half hour. Sleep duration on weekends or non-workdays were only collected in the 2017–18 cycle and were therefore not included in our analysis. National Sleep Foundation recommends 7–8 h of sleep for older adults and 7–9 h of sleep for adults, we therefore categorized usual sleep duration as 7–8 h (reference), <7 h, and >8 h, and in sensitivity analyses, we also considered 7–9 h (reference), <7 h and >9 h [34].

Other sleep characteristics served as our secondary exposures of interest. Participants reported their frequency of snoring and frequency of snorting or stopping breathing while sleeping in the past 12 months as never, rarely (1–2 nights/week), occasionally (3–4 nights/week) and frequently (≥5 nights/week). Participants were asked whether they ever told a doctor that they had trouble sleeping (Yes, No). Daytime sleepiness was assessed by self-reported frequency of feeling excessively or overly sleepy during the day in the past month, including never, rarely (1 time/month), sometimes (2–4 times/month), often (5–15 times/month), and almost always (16–30 times/month).

### 2.4. Other covariates

Demographic information was collected via self-report as a part of the in-home interview, including age in years, sex (Male, Female), race/ethnicity (Non-Hispanic White, Non-Hispanic Black, Hispanic, Other race) and education (Below high school, High school or equivalent, Above high school).

Smoking status was defined according to two questions: (1) “Have you smoked at least 100 cigarettes in entire life?” (Yes, No); (2) “Do you now smoke cigarettes?” (Every day, Some days, Not at all). Participants were categorized as never smoker if they answered “No” to the first question, former smoker if they answered “Yes” to the first question but “Not at all” to the second question and current smoker if they answered “Yes” to the first question and “Every day” or “Some days” to the second question. We defined heavy drinkers (Ever, Never) as males who ever had a time in their life having ≥5 drinks of alcohol almost every day or females who ever had ≥4 drinks of alcohol. Ever heavy drinkers were not further categorized as former and current drinkers due to small number of current drinkers. Occupational noise exposure (Yes, No) was defined as having a job exposure to loud noise for ≥4 h per day and several days per week. Off-work noise exposure (Yes, No) was defined as having noise exposure outside of work for ≥10 h per week.

Body mass index (BMI) was calculated from measured height and weight and analyzed continuously in kg/m^2^. Hypertension and diabetes (Yes, No) were defined according to self-reported diagnoses by physicians.

### 2.5. Statistical analysis

Characteristics of the participants were described and compared using one-way analysis of variance (ANOVA) for continuous variables and Pearson’s chi-squared test for categorical variables.

According to the NHANES analytic guidelines, to obtain nationally representative estimates, the NHANES sampling design was taken into account in all the multivariable-adjusted analyses [35]. To appropriately combine the 2015–16 and 2017–18 cycles of the NHANES, we constructed four-year weights for 2015–18 by averaging the 2015–16 and 2017–18 two-year sample weights. We used multivariable-adjusted linear regression to estimate differences in better-ear PTA associated with each sleep characteristic. We also used multivariable-adjusted Poisson regression to estimate prevalence ratios (PR) of having hearing loss associated with each sleep characteristic.

Given that both sleep and hearing change with age, the association between sleep and hearing among middle-aged and older adults might be modified by age [1,11]. We included an interaction term between sleep characteristic (categorical) and age (continuous in years) in all the models. To facilitate interpretation, we centered the age variable respectively at 50, 60 and 70 years to obtain age-specific estimates of the association.

Primary models adjusted for age, sex, race/ethnicity, education, smoking, drinking, occupational and off-work noise exposure. BMI, hypertension, and diabetes are potential mediators of the sleep-hearing associations as prior literature suggests that sleep disturbances might exacerbate cardiovascular factors, which have been linked to hearing loss [36,37]. Therefore, we additionally adjusted for these factors in secondary models.

All analyses were conducted using Stata, version 17.0 (StataCorp LLC, College Station, Texas) and a two-sided *P*-value <0.05 was considered statistically significant.

## 3. Results

Among 3100 participants aged over 40 years (mean age [standard deviation]: 60 [12] years, 51 % female, 22 % non-Hispanic Black), 1449 (47 %) participants reported usual sleep duration of 7–8 h on weekdays or workdays, 733 (24 %) slept <7 h and 918 (30 %) slept >8 h. Participants with usual sleep duration >8 h were older, more likely to self-identify as female and non-Hispanic White, less educated, more likely to have hypertension and worse audiometric hearing while participants who reported sleeping <7 h had higher BMI, were more likely to be current smokers and to report off-work noise exposure (Table 1).

A total of 754 (24 %) participants had hearing loss. When compared to 2346 (76 %) participants with normal hearing, participants with hearing loss were more likely to report longer usual sleep duration on weekdays or workdays (mean: 7.9 vs. 7.6 h) and a higher frequency of feeling overly sleepy during the day (almost always: 11% vs. 7 %) (Table 2).

**Fig. 1.**
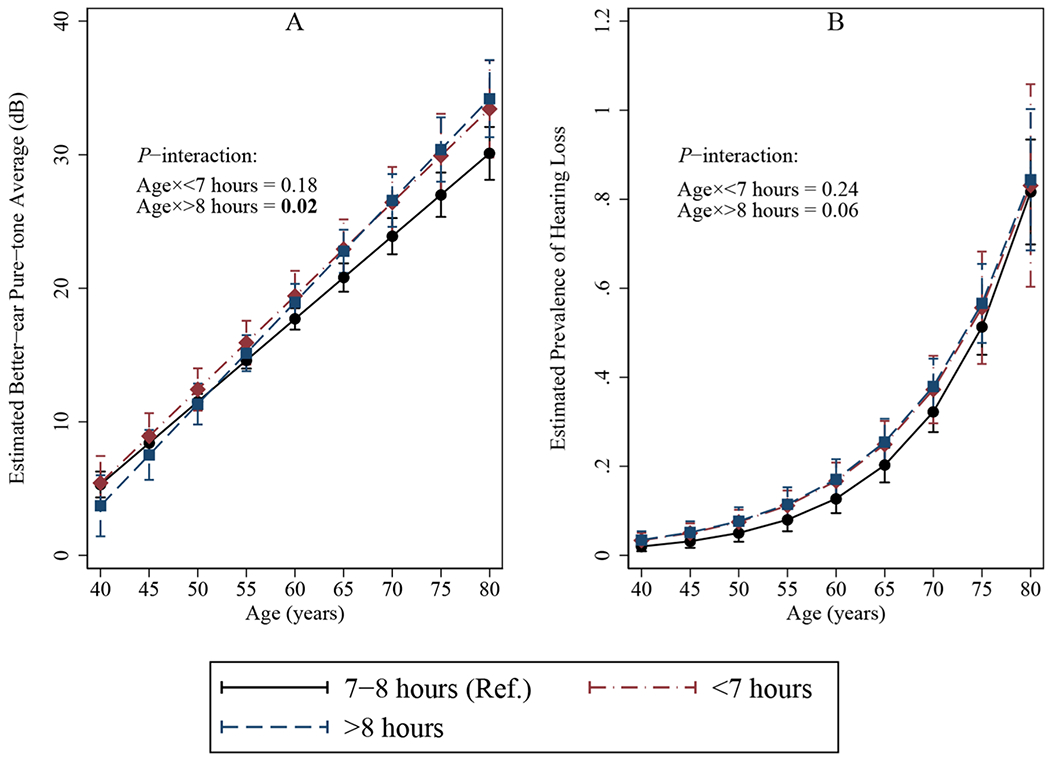
Multivariable-adjusted Associations of Usual Sleep Duration on Weekdays or Workdays with (A) Better-ear Pure-tone Average; (B) Prevalence of Hearing Loss; by Age, The National Health and Nutrition Examination Survey (NHANES) 2015–18. Multivariable-adjusted linear regression with better-ear pure-tone average as the outcome or multivariable-adjusted Poisson regression with hearing loss (Yes/No) as the outcome. An interaction term between usual sleep duration and continuous age in years was also included. Models adjusted for age, sex, race/ethnicity, education, smoking, drinking, occupational and off-work noise exposure.

When compared to participants reporting usual sleep duration between 7 and 8 h, participants sleeping <7 h and >8 h had similar PTA at younger ages but worse PTA at older ages and the differences in PTA were greater with increasing age, particularly among participants sleeping >8 h (*P*-interaction: Age×<7 h = 0.18; Age×>8 h = 0.02) (Fig. 1, Panel A). Specifically, it was estimated that at age 50, no significant differences in PTA by sleep duration was found; at age 60, borderline associations were found (<7 h: estimate = 1.72 dB, 95 % confidence interval [CI], −0.21, 3.66; >8 h: estimate = 1.25 dB, 95 % CI, −0.01, 2.51); at age 70, a significant association was found (<7 h: estimate = 2.52 dB, 95 % CI, −0.27, 5.31; >8 h: estimate = 2.67 dB, 95 % CI, 0.56, 4.79) (Table 3, Model 1). Additional adjustment for cardiovascular factors showed similar results; participants with sleep duration >8 h showed significantly worse PTA at age 70 (estimate = 2.69 dB, 95 % CI, 0.56, 4.83) (Table 3, Model 2). Using alternative cut-off points for sleep duration, a similar trend of greater impact of sleep duration on hearing with increasing age was found. Comparing to participants sleeping 7–9 h, significantly worse PTA was found among those sleeping >9 h (estimate = 3.46 dB, 95 % CI: 0.56, 6.36) whereas the association among those sleeping <7 h was not significant (estimate = 1.87 dB, 95 % CI: −1.27, 5.01) at age 70 (Table S2).

When considering clinically significant hearing loss, greater impact of long sleep duration on PTA with increasing age did not translate to greater impact on prevalence of hearing loss (Fig. 1, Panel B). We found slightly weaker prevalence ratios of hearing loss associated with long sleep duration with increasing age (*P*-interaction: Age×<7 h = 0.24; Age×>8 h = 0.06) (Table S3). Sleeping >8 h was still associated with higher prevalence of hearing loss comparing to those sleeping 7–8 h (Age 50: PR=1.53, 95 % CI, 1.11, 2.11; Age 60: PR=1.34, 95 % CI, 1.08, 1.66; Age 70: PR=1.18, 95 % CI, 1.01, 1.38) (Table S3).

We did not find associations of other sleep characteristics (snoring, snorting or stopping breathing, trouble sleeping, daytime sleepiness) with hearing and the associations were not significantly modified by age. Participants reported occasional snorting or stopping breathing had worse hearing comparing to those reported never (Age 50: estimate = 2.19 dB, 95 % CI, −0.11, 4.49; Age 60: estimate = 3.84 dB, 95 % CI, 0.25, 7.43; Age 70: estimate = 5.48 dB, 95 % CI, −0.19, 11.16) and the association was stronger with increasing age (*P*-interaction=0.18) (Model 1: Table 4; Model 2: Table S4). However, we did not observe a consistent trend of worse hearing with higher frequencies of snorting or stopping breathing. In addition, these associations were not significant with prevalence of clinically significant hearing loss (Table S5).

Participants reported almost always feeling overly sleepy had significantly higher prevalence of hearing loss when compared to those reported never feeling overly sleepy (Age 50: PR = 2.54, 95 % CI, 1.19, 5.42; Age 60: PR = 1.92, 95 % CI, 1.17, 3.14; Age 70: PR = 1.44, 95 % CI, 1.05, 1.98) and the association was weaker with increasing age (*P*-interaction=0.07). However, we did not observe a consistent trend of worse hearing with increasing frequency of feeling overly sleepy (Table S5). No significant associations were found when continuous PTA was modeled as the outcome.

## 4. Discussion

In this nationally representative sample of middle-aged and older adults (age: 60 ± 12 years, 51 % female, 22 % Black), we found that, compared to participants reporting sleep duration of 7–8 h, those reporting >8 h of sleep had significantly worse audiometric hearing in older ages, whereas sleeping <7 h showed borderline significance after adjusting for demographic, lifestyle and cardiovascular factors. The association between short/long sleep duration and worse audiometric hearing became stronger with increasing age. Other sleep characteristics (snoring, snorting or stopping breathing, trouble sleeping, daytime sleepiness) were not consistently associated with hearing.

Our previous analysis in NHANES 2005–06 reported an association between long sleep duration and high-frequency hearing loss [17]. However, we were only able to examine the association among participants aged 70 years and older as the audiometry examination was only administered to a subset of participants. With the combination of two recent NHANES cycles for the current study, we found that sleep durations <7 h and >8 h were associated with worse audiometric hearing. In addition, with the examination of age × sleep interaction, we also reported a stronger association between sleep duration and hearing with increasing age. This indicates that sleep duration may have greater implications for hearing at older ages. Sleep duration at younger ages might be complicated by employment instead of reflecting underlying health status. As usual sleep duration on weekends was only asked in NHANES 2017–18, we were not able to incorporate this question. For older adults, usual sleep duration on weekdays and on weekends might not be that different, but for middle-aged adults, our finding might be obscured when only examining sleep duration on weekdays. When we modeled prevalence of clinically significant hearing loss instead of differences in PTA, we found slightly weaker associations between sleep duration and hearing loss. Caution should be taken when interpreting this contradictory finding. The prevalence of hearing loss increases dramatically with increasing age, though the estimated association was stronger at age 50, the confidence interval was also wider.

When additionally adjusting for cardiovascular factors, the associations were not attenuated, suggesting that cardiovascular factors might not be a major confounder of the association between sleep duration and hearing loss. Prior research for this specific association is lacking and there is no well-supported hypothesis specific for sleep duration and hearing loss. Further research with brain magnetic resonance imaging might consider other possible mechanisms like neurological changes in brain structure and function associated with extreme sleep duration that can also impact auditory processing.

Similar to our previous study in NHANES, we did not find consistent associations between frequencies of snoring and snorting or stopping breathing with hearing, though this association was most widely supported by previous studies [16,19,23–25,28]. And impaired cochlear function due to hypoxia resulting from breathing abnormalities is a plausible underlying pathway that has been proposed [12]. In our secondary models with additional adjustment for cardiovascular factors, estimated associations were slightly attenuated, which supports the hypothesis. But we only included self-reported diagnoses of hypertension and diabetes, therefore, other cardiovascular factors and subclinical changes were not captured. In addition to differences in characteristics of participants (clinical vs. population-based cohort), the inconsistent finding could be due to the self-reported nature of the sleep questions in NHANES and participants are likely to under-report their frequencies of snoring and snorting or stopping breathing, which would lead to underestimated associations. Our analysis in the Atherosclerosis Risk in Communities-Sleep Heart Health Study (ARIC-SHHS) found 2.51 dB worse audiometric hearing associated with SDB assessed by polysomnography, and consistent with our hypothesis, the association was attenuated by adjustment for cardiovascular factors [16]. Taken together, self-reported SDB is likely not sufficient and may miss occult SDB. To better understand the association between SDB and hearing loss and the potential roles of cardiovascular factors in the association, studies with polysomnography-assessed SDB are needed.

We found an association between daytime sleepiness and higher prevalence of hearing loss and the association was stronger at younger ages, though this finding was not consistent. One possible explanation is that younger adults were more likely to report higher frequencies of feeling overly sleepy during the day due to multiple work and family responsibilities, however, the explanation is not sufficient given inconsistent trend in proportions of participants reported almost always feeling overly sleepy (40–49 years: 6 %; 50–59 years: 10 %; 60–69 years: 5 %; 70–79 years: 9 %). Our ARIC-SHHS analysis found 3.35 dB worse audiometric hearing associated with excessive daytime sleepiness, as assessed by the Epworth Sleepiness Scale [16]. Feeling overly sleepy might represent overall health status, such that participants who reported more frequent sleepiness might have underlying disease processes that can co-occur or lead to hearing loss. Inconsistencies between these findings and those of the current study may be due to a single item in the current study vs. a validated measure like the Epworth Sleepiness Scale.

Longitudinal evidence of the associations between sleep disturbances and hearing loss is still limited. Findings from recent studies suggest sleep disturbances might be a novel risk factor causally contributing to hearing loss. A study in the UK Biobank cohort with a median follow-up of four years reported that a greater number of sleep complaints was associated with a higher risk of incident self-reported hearing loss; however, there was no association between sleep duration and hearing loss [15]. Our ARIC-SHHS analysis among 719 White participants reported associations of longer sleep duration, shorter rapid eye movement sleep, SDB, and excessive daytime sleepiness with late-life hearing loss assessed 20 years later [16]. Future longitudinal studies are needed to clarify the potential causal relationships between sleep disturbances and hearing loss.

Our study has a number of strengths. First, we examined the associations between sleep characteristics and hearing in a nationally representative sample, which is more generalizable than occupational and clinical cohorts. Second, we included multiple sleep measures to capture different aspects of sleep. Last, we expanded upon our previous NHANES study by having a wider age range of participants and studied how age modifies the sleep-hearing association, contributing to the existing literature by taking a life course perspective. However, this study is cross-sectional and we cannot establish temporality between our sleep and hearing measures. Also, sleep characteristics were self-reported and objective sleep measures obtained from polysomnography or actigraphy were not available in our study. We were unable to measure sleep stages or quantify SDB. This is a limitation because slow-wave sleep may help prevent amyloid deposition, and SDB is linked to Alzheimer’s disease biomarkers and dementia risk. Moreover, hypoxia due to SDB and related snoring are important risk factors for hearing loss. Studies with objective sleep measures will be essential to better elucidate the mechanisms underlying our findings. Although it can be challenging to accomplish this in a large nationally representative sample, advances in wearable devices are making this increasingly feasible. Cognitive measures were not available in these two cycles of NHANES, though it is likely that there are participants with cognitive impairment or underlying brain pathologies that can affect sleep or their self-report of sleep disturbances in the study, valid audiometric hearing measures can be obtained among people with cognitive impairment [38, 39].

Accumulating evidence suggests an association between sleep disturbances and hearing loss. Our findings have important implications for screening and management of sleep disturbances. In addition to its benefits in preventing cardiovascular diseases and cognitive impairments, screening for and management of sleep disturbances might contribute to hearing loss prevention, if sleep disturbances are risk factors for hearing loss, as we hypothesized [3,40]. Also, sleep disturbances can be effectively and safely managed [41]. We observed stronger sleep duration – hearing loss association with older age, management of sleep disturbances might therefore be particularly important for older adults commonly living with comorbid conditions. In addition, because sleep disturbances and hearing loss are both established risk factors for adverse aging-related outcomes including dementia, the observed sleep-hearing association might imply their common co-occurrence among older adults and potential interaction to jointly contribute to adverse outcomes. Screening for and management of sleep disturbances might provide additional benefits by helping prevent hearing loss, including dementia prevention.

In summary, this study found cross-sectional associations of short/long sleep duration with hearing loss that are stronger among older adults. Sleep characteristics might be risk factors for hearing loss and their contribution to hearing loss can differ by age. Further evidence is needed to clarify whether sleep disturbances are risk factors causally contributing to hearing loss or a marker of hearing loss.

## Supplementary Material

1

## Figures and Tables

**Table 1:** Demographic, Lifestyle and Clinical Characteristics of Participants by Usual Sleep Duration on Weekdays or Workdays, The National Health and Nutrition Examination Survey (NHANES) 2015–18.

	Total *N* = 3100	Usual Sleep Duration on Weekdays or Workdays (Hours)^a^			*P*-value^b^
		<7 *N* = 733	7–8 *N* = 1449	> 8 *N* = 918	
**Age (year), Mean (SD)**	59.7 (12.1)	58.1 (11.5)	58.8 (12.0)	62.5 (12.2)	<0.001
**Female, N (%)**	1590 (51.3)	321 (43.8)	731 (50.4)	538 (58.6)	<0.001
**Race/Ethnicity, N (%)**					<0.001
Non-Hispanic White	1169 (37.7)	206 (28.1)	573 (39.5)	390 (42.5)	
Non-Hispanic Black	666 (21.5)	221 (30.2)	296 (20.4)	149 (16.2)	
Hispanic	859 (27.7)	200 (27.3)	382 (26.4)	277 (30.2)	
Other	406 (13.1)	106 (14.5)	198 (13.7)	102 (11.1)	
**Education, N (%)**					<0.001
< High School	738 (23.8)	175 (23.9)	297 (20.5)	266 (29.0)	
High School or Equivalent	705 (22.7)	182 (24.8)	306 (21.1)	217 (23.6)	
> High School	1657 (53.5)	376 (51.3)	846 (58.4)	435 (47.4)	
**Smoking, N (%)**					0.02
Never	1664 (53.7)	361 (49.2)	811 (56.0)	492 (53.6)	
Former	886 (28.6)	217 (29.6)	409 (28.2)	260 (28.3)	
Current	550 (17.7)	155 (21.1)	229 (15.8)	166 (18.1)	
**Heavy Drinker, N (%)**	483 (15.6)	125 (17.1)	211 (14.6)	147 (16.0)	0.29
**Occupational Noise Exposure, N (%)**	1113 (35.9)	283 (38.6)	502 (34.6)	328 (35.7)	0.19
**Off-Work Noise Exposure, N (%)**	391 (12.6)	113 (15.4)	171 (11.8)	107 (11.7)	0.03
**Body Mass Index (kg/m^2^), Mean (SD)**	30.0 (6.8)	30.6 (7.4)	29.7 (6.7)	29.9 (6.6)	0.01
**Hypertension, N (%)**	1513 (48.8)	369 (50.3)	652 (45.0)	492 (53.6)	<0.001
**Diabetes, N (%)**	789 (25.5)	189 (25.8)	343 (23.7)	257 (28.0)	0.06
**Better-ear Pure-tone Average**^c^ **(dB), Mean (SD)**	18.8 (14.1)	18.1 (13.5)	17.4 (13.2)	21.5 (15.6)	<0.001
**Hearing Loss**^d^, **N (%)**	754 (24.3)	167 (22.8)	295 (20.4)	292 (31.8)	<0.001

Abbreviations: SD, standard deviation; dB, decibels.

a Participants were asked to report their usual time to sleep and usual wake time during main sleepingg period (night or day) for workdays or weekdays and their usual hours of sleep were calculated from these two questions.

b *P*-values were calculated by ANOVA for continuous variables and Pearson chi-squared test for categorical variables.

c Audiometric hearing thresholds at 0.5, 1, 2 and 4 kilohertz in better-hearing ear were averaged.

d Hearing loss was defined as four-frequency (0.5, 1, 2 and 4 kilohertz) pure-tone average in better hearing ear >25 dB.

**Table 2:** Sleep Characteristics of Participants by Hearing Loss, The National Health and Nutrition Examination Survey (NHANES) 2015–18.

	Total *N* = 3100	Normal Hearing *N* = 2346	Hearing Loss^a^ *N* = 754	*P*-value^b^
**Usual Sleep Duration on Weekdays or Workdays (Hour), Mean (SD)**	7.6 (1.5)	7.6 (1.5)	7.9 (1.7)	<0.001
**How often do you snore, N (%)**				0.07
Never	652 (21.0)	478 (20.4)	174 (23.1)	
Rarely	666 (21.5)	524 (22.3)	142 (18.8)	
Occasionally	612 (19.7)	482 (20.5)	130 (17.2)	
Frequently	911 (29.4)	696 (29.7)	215 (28.5)	
Missing	259 (8.4)	166 (7.1)	93 (12.3)	
**How often do you snort or stop breathing, N (%)**				0.20
Never	2040 (65.8)	1564 (66.7)	476 (63.1)	
Rarely	408 (13.2)	308 (13.1)	100 (13.3)	
Occasionally	237 (7.6)	180 (7.7)	57 (7.6)	
Frequently	188 (6.1)	131 (5.6)	57 (7.6)	
Missing	227 (7.3)	163 (6.9)	64 (8.5)	
**Ever told doctor had trouble sleeping, N (%)**	983 (31.7)	729 (31.1)	254 (33.7)	0.18
**How often feel overly sleepy during day, N (%)**				0.003
Never	587 (18.9)	454 (19.4)	133 (17.6)	
Rarely	748 (24.1)	584 (24.9)	164 (21.8)	
Sometimes	1004 (32.4)	763 (32.5)	241 (32.0)	
Often	509 (16.4)	378 (16.1)	131 (17.4)	
Almost always	252 (8.1)	167 (7.1)	85 (11.3)	

Abbreviations: SD, standard deviation.

a Hearing loss was defined as four-frequency (0.5, 1, 2 and 4 kilohertz) pure-tone average in better hearing ear >25 decibels.

b *P*-values were calculated by ANOVA for continuous variables and Pearson chi-squared test for categorical variables.

**Table 3:** Multivariable-adjusted ^a^ Associations between Usual Sleep Duration on Weekdays or Workdays and Audiometric Hearing by Age, The National Health and Nutrition Examination Survey (NHANES) 2015–18.

	Age 50		Age 60		Age 70		
Model 1^b^	Estimate (95 % CI)	*P*-value	Estimate (95 % CI)	*P*-value	Estimate (95 % CI)	*P*-value	*P*-interaction

7–8 h	Ref.	–	Ref.	–	Ref.	–	–
<7 h	0.92 (−0.70, 2.54)	0.26	1.72 (−0.21, 3.66)	0.08	2.52 (−0.27, 5.31)	0.07	0.18
>8 h	–0.17 (−1.34, 0.99)	0.76	1.25 (−0.01, 2.51)	0.05	**2.67 (0.56, 4.79)**	**0.02**	**0.02**
**Model 2** ^c^	**Estimate (95 % CI)**	***P*-value**	**Estimate (95 % CI)**	***P*-value**	**Estimate (95 % CI)**	***P*-value**	***P*-interaction**
7–8 h	Ref.	–	Ref.	–	Ref.	–	–
<7 h	0.82 (−0.79, 2.42)	0.31	1.59 (−0.32, 3.49)	0.10	2.36 (−0.35, 5.07)	0.09	0.18
>8 h	−0.31 (−1.46, 0.84)	0.59	1.19 (−0.05, 2.44)	0.06	**2.69 (0.56, 4.83)**	**0.02**	**0.01**

Abbreviations: Ref, reference; CI, confidence interval.

a Multivariable-adjusted linear regression with better-ear pure-tone average as the outcome and usual sleep duration as the exposure. An interaction term between sleep characteristic and continuous age in years was also included and age was centered at 50, 60 and 70 years respectively to obtain age-specific estimates.

b Model 1 adjusted for age, sex, race/ethnicity, education, smoking, drinking, occupational and off-work noise exposure.

c Model 2 adjusted for age, sex, race/ethnicity, education, smoking, drinking, occupational and off-work noise exposure, body mass index, hypertension, and diabetes.

**Table 4:** Multivariable-adjusted ^a^ Differences in Better-ear Pure-tone Average by Other Sleep Characteristics and Age, The National Health and Nutrition Examination Survey (NHANES) 2015–18.

	Age 50		Age 60		Age 70		*P*-interaction
	Estimate (95 % CI)	*P*-value	Estimate (95 % CI)	*P*-value	Estimate (95 % CI)	*P*-value	
**Frequency of Snoring**							
Never	Ref.	–	Ref.	–	Ref.	–	–
Rarely	1.02 (−0.35, 2.40)	0.14	0.39 (−1.62, 2.39)	0.70	−0.25 (−3.29, 2.78)	0.87	0.30
Occasionally	−0.93 (−2.22, 0.36)	0.15	−1.47 (−2.97, 0.02)	0.05	−2.02 (−4.46, 0.42)	0.10	0.38
Frequently	0.15 (−1.07, 1.37)	0.80	−0.40 (−1.64, 0.85)	0.52	−0.94 (−2.98, 1.10)	0.35	0.33
***P***-**trend**	0.43		0.13		0.18		0.44
**Frequency of Snorting or Stopping Breathing**							
Never	Ref.	–	Ref.	–	Ref.	–	–
Rarely	−1.02 (−3.28, 1.24)	0.36	−0.19 (−2.66, 2.27)	0.87	0.64 (−3.04, 4.31)	0.73	0.35
Occasionally	2.19 (−0.11, 4.49)	0.06	**3.84 (0.25, 7.43)**	**0.04**	5.48 (−0.19, 11.16)	0.06	0.18
Frequently	0.34 (−1.12, 1.80)	0.64	0.31 (−1.47, 2.09)	0.72	0.29 (−2.70, 3.27)	0.85	0.97
***P*-trend**	0.27		0.13		0.15		0.32
**Trouble Sleeping**							
No	Ref.	–	Ref.	–	Ref.	–	–
Yes	0.42 (−1.13, 1.96)	0.59	0.33 (−1.33, 1.98)	0.69	0.23 (−2.14, 2.61)	0.84	0.87
**Frequency of Feeling Overly Sleepy**							
Never	Ref.	–	Ref.	–	Ref.	–	–
Rarely	0.00 (−1.34, 1.35)	0.99	0.54 (−1.44, 2.52)	0.58	1.08 (−2.30, 4.45)	0.52	0.51
Sometimes	−1.07 (−2.38, 0.25)	0.11	−0.54 (−2.37, 1.29)	0.55	−0.01 (−2.67, 2.65)	0.99	0.30
Often	−0.62 (−2.18, 0.94)	0.42	0.38 (−1.71, 2.46)	0.71	1.38 (−1.89, 4.65)	0.40	0.18
Almost always	1.76 (−1.12, 4.64)	0.22	2.58 (−0.39, 5.55)	0.09	3.41 (−0.55, 7.37)	0.09	0.35
***P*-trend**	0.85		0.36		0.21		0.18

Abbreviations: Ref, reference; CI, confidence interval.

a Multivariable-adjusted linear regression with better-ear pure-tone average as the outcome and each sleep characteristic as the exposure. An interaction term between sleep characteristic and continuous age in years was also included and age was centered at 50, 60 and 70 years respectively to obtain age-specific estimates. Models adjusted for age, sex, race/ethnicity, education, smoking, drinking, occupational and off-work noise exposure.

## Data Availability

Publicly available datasets were used in this study. These can be found in CDC/National Center for Health Statistics at https://www.cdc.gov/nchs/nhanes/index.htm.
