# Supplementary material for: Sleep characteristics and hearing loss in middle-aged and older adults: The National Health and Nutrition Examination Survey 2015–2018
Source: Sleep Epidemiol. Author manuscript; Available in PMC 2025 Jan 30. (PMC11781046; doi:10.1016/j.sleepe.2024.100082)
Supplement: 1 [file NIHMS2041524-supplement-1.docx]

**Supplemental Material**

[Table S1. Demographic, Lifestyle and Clinical Characteristics of the Participants by Included vs. Excluded in the Study Population, The National Health and Nutrition Examination Survey (NHANES) 2015-18 2](#_Toc123909111)

[Table S2. Multivariable-adjusted Associations between Usual Sleep Duration on Weekdays or Workdays Using Alternative Categorization and Audiometric Hearing by Age, The National Health and Nutrition Examination Survey (NHANES) 2015-18 3](#_Toc123909112)

[Table S3. Multivariable-adjusted Prevalence Ratios (PR) of Hearing Loss by Usual Sleep Duration on Weekdays or Workdays and Age, The National Health and Nutrition Examination Survey (NHANES) 2015-18 4](#_Toc123909113)

[Table S4. Multivariable-adjusted Differences in Better-ear Pure-tone Average by Other Sleep Characteristics and Age, The National Health and Nutrition Examination Survey (NHANES) 2015-18 5](#_Toc123909114)

[Table S5. Multivariable-adjusted Prevalence Ratios (PR) of Hearing Loss by Other Sleep Characteristics and Age, The National Health and Nutrition Examination Survey (NHANES) 2015-18 6](#_Toc123909115)

# **Table S1. Demographic, Lifestyle and Clinical Characteristics of Participants by Included vs. Excluded in the Study Population, The National Health and Nutrition Examination Survey (NHANES) 2015-18**

|  | **Total**  **N=3,679** | **Excluded**  **N=579** | **Included**  **N=3,100** | ***P*-value ^a^** |
| --- | --- | --- | --- | --- |
| **Age (year), Mean (SD)** | 60.1 (12.3) | 61.8 (13.3) | 59.7 (12.1) | <0.001 |
| **Female, N (%)** | 1905 (51.8) | 315 (54.4) | 1590 (51.3) | 0.17 |
| **Race/Ethnicity, N (%)** | | | | <0.001 |
| Non-Hispanic White | 1331 (36.2) | 162 (28.0) | 1169 (37.7) |  |
| Non-Hispanic Black | 802 (21.8) | 136 (23.5) | 666 (21.5) |  |
| Hispanic | 1016 (27.6) | 157 (27.1) | 859 (27.7) |  |
| Other | 530 (14.4) | 124 (21.4) | 406 (13.1) |  |
| **Education, N (%)** | | | | <0.001 |
| < High School | 927 (25.2) | 189 (33.0) | 738 (23.8) |  |
| High School or Equivalent | 820 (22.3) | 115 (20.1) | 705 (22.7) |  |
| > High School | 1926 (52.4) | 269 (46.9) | 1657 (53.5) |  |
| **Smoking, N (%)** | | | | 0.19 |
| Never | 1995 (54.3) | 331 (57.4) | 1664 (53.7) |  |
| Former | 1031 (28.0) | 145 (25.1) | 886 (28.6) |  |
| Current | 651 (17.7) | 101 (17.5) | 550 (17.7) |  |
| **Heavy Drinker, N (%)** | 528 (15.8) | 45 (18.5) | 483 (15.6) | 0.23 |
| **Occupational Noise Exposure, N (%)** | 1299 (35.3) | 186 (32.3) | 1113 (35.9) | 0.10 |
| **Off-Work Noise Exposure, N (%)** | 451 (12.3) | 60 (10.4) | 391 (12.6) | 0.14 |
| **Body Mass Index (kg/m^2^), Mean (SD)** | 29.8 (6.8) | 28.6 (6.5) | 30.0 (6.8) | <0.001 |
| **Hypertension, N (%)** | 1800 (49.0) | 287 (49.8) | 1513 (48.8) | 0.65 |
| **Diabetes, N (%)** | 917 (24.9) | 128 (22.2) | 789 (25.5) | 0.10 |

Abbreviations: SD, standard deviation.

^a^ *P*-values were calculated by ANOVA for continuous variables and Pearson chi-squared test for categorical variables.

# **Table S2. Multivariable-adjusted ^a^ Associations between Usual Sleep Duration on Weekdays or Workdays Using Alternative Categorization and Audiometric Hearing by Age, The National Health and Nutrition Examination Survey (NHANES) 2015-18**

|  | **Age 50** | | **Age 60** | | **Age 70** | |  |
| --- | --- | --- | --- | --- | --- | --- | --- |
| **Model 1 ^b^** | **Estimate (95% CI)** | ***P*-value** | **Estimate (95% CI)** | ***P*-value** | **Estimate (95% CI)** | ***P*-value** | ***P*-interaction** |
| 7-9 hours | Ref. | - | Ref. | - | Ref. | - | - |
| <7 hours | 1.01 (-0.72, 2.74) | 0.24 | 1.44 (-0.73, 3.61) | 0.19 | 1.87 (-1.27, 5.01) | 0.23 | 0.51 |
| >9 hours | 0.14 (-1.63, 1.91) | 0.87 | 1.80 (-0.11, 3.71) | 0.06 | **3.46 (0.56, 6.36)** | **0.02** | **0.03** |
| **Model 2 ^c^** | **Estimate (95% CI)** | ***P*-value** | **Estimate (95% CI)** | ***P*-value** | **Estimate (95% CI)** | ***P*-value** | ***P*-interaction** |
| 7-9 hours | Ref. | - | Ref. | - | Ref. | **-** | **-** |
| <7 hours | 0.91 (-0.79, 2.62) | 0.25 | 1.29 (-0.84, 3.41) | 0.23 | 1.66 (-1.40, 4.72) | 0.28 | 0.55 |
| >9 hours | -0.20 (-2.01, 1.62) | 0.83 | 1.51 (-0.40, 3.42) | 0.12 | **3.21 (0.26, 6.16)** | **0.03** | **0.03** |

Abbreviations: Ref, reference; CI, confidence interval.

^a^ Multivariable-adjusted linear regression with better-ear pure-tone average as the outcome and usual sleep duration as the exposure. An interaction term between sleep characteristic and continuous age in years was also included and age was centered at 50, 60 and 70 years respectively to obtain age-specific estimates.

^b^ Model 1 adjusted for age, sex, race/ethnicity, education, smoking, drinking, occupational and off-work noise exposure.

^c^ Model 2 adjusted for age, sex, race/ethnicity, education, smoking, drinking, occupational and off-work noise exposure, body mass index, hypertension and diabetes.

# **Table S3. Multivariable-adjusted ^a^ Prevalence Ratios (PR) of Hearing Loss by Usual Sleep Duration on Weekdays or Workdays and Age, The National Health and Nutrition Examination Survey (NHANES) 2015-18**

|  | **Age 50** | | **Age 60** | | **Age 70** | |  |
| --- | --- | --- | --- | --- | --- | --- | --- |
| **Model 1 ^b^** | **PR (95% CI)** | ***P*-value** | **PR (95% CI)** | ***P*-value** | **PR (95% CI)** | ***P*-value** | ***P*-interaction** |
| 7-8 hours | Ref. | - | Ref. | - | Ref. | - | - |
| <7 hours | 1.49 (0.88, 2.51) | 0.13 | 1.31 (0.92, 1.86) | 0.12 | 1.16 (0.89, 1.50) | 0.26 | 0.24 |
| >8 hours | **1.53 (1.11, 2.11)** | **0.01** | **1.34 (1.08, 1.66)** | **0.01** | **1.18 (1.01, 1.38)** | **0.04** | 0.06 |
| **Model 2 ^c^** | **PR (95% CI)** | ***P*-value** | **PR (95% CI)** | ***P*-value** | **PR (95% CI)** | ***P*-value** | ***P*-interaction** |
| 7-8 hours | Ref. | - | Ref. | - | Ref. | **-** | **-** |
| <7 hours | 1.48 (0.87, 2.52) | 0.14 | 1.29 (0.91, 1.84) | 0.14 | 1.13 (0.89, 1.44) | 0.31 | 0.22 |
| >8 hours | **1.53 (1.11, 2.09)** | **0.01** | **1.34 (1.08, 1.65)** | **0.01** | **1.17 (1.00, 1.37)** | **0.05** | 0.06 |

Abbreviations: Ref, reference; CI, confidence interval.

^a^ Multivariable-adjusted Poisson regression with hearing loss (Yes/No) as the outcome and usual sleep duration as the exposure. An interaction term between sleep characteristic and continuous age in years was also included and age was centered at 50, 60 and 70 years respectively to obtain age-specific estimates.

^b^ Model 1 adjusted for age, sex, race/ethnicity, education, smoking, drinking, occupational and off-work noise exposure.

^c^ Model 2 adjusted for age, sex, race/ethnicity, education, smoking, drinking, occupational and off-work noise exposure, body mass index, hypertension and diabetes.

# **Table S4. Multivariable-adjusted ^a^ Differences in Better-ear Pure-tone Average by Other Sleep Characteristics and Age, The National Health and Nutrition Examination Survey (NHANES) 2015-18**

|  | **Age 50** | | **Age 60** | | **Age 70** | | ***P*-interaction** |
| --- | --- | --- | --- | --- | --- | --- | --- |
|  | **Estimate (95% CI)** | ***P*-value** | **Estimate (95% CI)** | ***P*-value** | **Estimate (95% CI)** | ***P*-value** |  |
| **Frequency of Snoring** | | | | | | | |
| Never | Ref. | - | Ref. | - | Ref. | - | - |
| Rarely | 1.02 (-0.36, 2.40) | 0.14 | 0.48 (-1.52, 2.47) | 0.63 | -0.07 (-3.10, 2.97) | 0.96 | 0.39 |
| Occasionally | -1.14 (-2.55, 0.26) | 0.11 | -1.47 (-3.02, 0.08) | 0.06 | -1.80 (-4.27, 0.67) | 0.15 | 0.60 |
| Frequently | -0.45 (-1.97, 1.07) | 0.55 | -0.86 (-2.33, 0.61) | 0.24 | -1.27 (-3.48, 0.95) | 0.25 | 0.49 |
| ***P*-trend** | 0.13 | | **0.05** | | 0.11 | | 0.66 |
| **Frequency of Snorting or Stopping Breathing** | | | | | | | |
| Never | Ref. | - | Ref. | - | Ref. | - | - |
| Rarely | -1.17 (-3.42, 1.09) | 0.30 | -0.18 (-2.62, 2.25) | 0.88 | 0.80 (-2.71, 4.31) | 0.65 | 0.24 |
| Occasionally | 1.94 (-0.51, 4.38) | 0.12 | **3.64 (0.00, 7.28)** | **0.05** | 5.34 (-0.28, 10.97) | 0.06 | 0.15 |
| Frequently | -0.39 (-2.06, 1.28) | 0.63 | -0.51 (-2.44, 1.41) | 0.59 | -0.63 (-3.80, 2.54) | 0.69 | 0.88 |
| ***P*-trend** | 0.65 | | 0.31 | | 0.26 | | 0.32 |
| **Trouble Sleeping** | | | | | | | |
| No | Ref. | - | Ref. | - | Ref. | - | - |
| Yes | 0.14 (-1.34, 1.62) | 0.85 | 0.08 (-1.57, 1.73) | 0.92 | 0.02 (-2.40, 2.44) | 0.99 | 0.92 |
| **Frequency of Feeling Overly Sleepy** | | | | | | | |
| Never | Ref. | - | Ref. | - | Ref. | - | - |
| Rarely | -0.20 (-1.52, 1.12) | 0.76 | 0.29 (-1.76, 2.34) | 0.77 | 0.78 (-2.73, 4.30) | 0.65 | 0.56 |
| Sometimes | **-1.33 (-2.65, -0.00)** | **0.05** | -0.79 (-2.72, 1.14) | 0.41 | -0.26 (-3.08, 2.56) | 0.85 | 0.31 |
| Often | -1.03 (-2.57, 0.50) | 0.18 | 0.01 (-2.10, 2.11) | 0.99 | 1.05 (-2.29, 4.39) | 0.52 | 0.17 |
| Almost always | 0.82 (-2.12, 3.76) | 0.57 | 1.69 (-1.38, 4.76) | 0.27 | 2.56 (-1.52, 6.65) | 0.21 | 0.33 |
| ***P*-trend** | 0.67 | | 0.69 | | 0.37 | | 0.15 |

Abbreviations: Ref, reference; CI, confidence interval.

^a^ Multivariable-adjusted linear regression with better-ear pure-tone average as the outcome and each sleep characteristic as the exposure. An interaction term between sleep characteristic and continuous age in years was also included and age was centered at 50, 60 and 70 years respectively to obtain age-specific estimates. Models adjusted for age, sex, race/ethnicity, education, smoking, drinking, occupational and off-work noise exposure, body mass index, hypertension and diabetes.

# **Table S5. Multivariable-adjusted ^a^ Prevalence Ratios (PR) of Hearing Loss by Other Sleep Characteristics and Age, The National Health and Nutrition Examination Survey (NHANES) 2015-18**

|  | **Age 50** | | **Age 60** | | **Age 70** | | ***P*-interaction** |
| --- | --- | --- | --- | --- | --- | --- | --- |
|  | **PR (95% CI)** | ***P*-value** | **PR (95% CI)** | ***P*-value** | **PR (95% CI)** | ***P*-value** |  |
| **Frequency of Snoring** | | | | | | | |
| Never | Ref. | - | Ref. | - | Ref. | - | - |
| Rarely | 1.43 (0.79, 2.58) | 0.23 | 1.30 (0.89, 1.89) | 0.16 | 1.18 (0.94, 1.48) | 0.14 | 0.44 |
| Occasionally | 0.77 (0.40, 1.47) | 0.41 | 0.87 (0.56, 1.35) | 0.52 | 0.99 (0.72, 1.35) | 0.94 | 0.33 |
| Frequently | 1.19 (0.70, 2.02) | 0.51 | 1.16 (0.84, 1.59) | 0.36 | 1.12 (0.95, 1.33) | 0.17 | 0.80 |
| ***P*-trend** | 0.84 | | 0.97 | | 0.51 | | 0.64 |
| **Frequency of Snorting or Stopping Breathing** | | | | | | | |
| Never | Ref. | - | Ref. | - | Ref. | - | - |
| Rarely | 1.34 (0.66, 2.70) | 0.41 | 1.23 (0.77, 1.97) | 0.38 | 1.13 (0.84, 1.53) | 0.40 | 0.54 |
| Occasionally | 1.77 (0.95, 3.30) | 0.07 | 1.51 (0.99, 2.31) | 0.06 | 1.29 (0.90, 1.86) | 0.16 | 0.26 |
| Frequently | 1.44 (0.74, 2.81) | 0.27 | 1.25 (0.78, 2.01) | 0.34 | 1.09 (0.76, 1.56) | 0.64 | 0.26 |
| ***P*-trend** | **0.04** | | 0.06 | | 0.25 | | 0.09 |
| **Trouble Sleeping** | | | | | | | |
| No | Ref. | - | Ref. | - | Ref. | - | - |
| Yes | 1.30 (0.82, 2.06) | 0.26 | 1.25 (0.92, 1.69) | 0.14 | **1.20 (1.01, 1.42)** | **0.04** | 0.65 |
| **Frequency of Feeling Overly Sleepy** | | | | | | | |
| Never | Ref. | - | Ref. | - | Ref. | - | - |
| Rarely | 1.06 (0.68, 1.64) | 0.79 | 1.10 (0.82, 1.47) | 0.53 | 1.14 (0.90, 1.43) | 0.27 | 0.71 |
| Sometimes | 0.74 (0.38, 1.46) | 0.37 | 0.85 (0.54, 1.35) | 0.48 | 0.98 (0.73, 1.30) | 0.86 | 0.24 |
| Often | 0.87 (0.54, 1.42) | 0.57 | 0.91 (0.65, 1.27) | 0.56 | 0.95 (0.76, 1.18) | 0.63 | 0.63 |
| Almost always | **2.54 (1.19, 5.42)** | **0.02** | **1.92 (1.17, 3.14)** | **0.01** | **1.44 (1.05, 1.98)** | **0.02** | 0.07 |
| ***P*-trend** | 0.23 | | 0.25 | | 0.40 | | 0.21 |

Abbreviations: Ref, reference; CI, confidence interval.

^a^ Multivariable-adjusted Poisson regression with hearing loss (Yes/No) as the outcome and each sleep characteristic as the exposure. An interaction term between sleep characteristic and continuous age in years was also included and age was centered at 50, 60 and 70 years respectively to obtain age-specific estimates. Models adjusted for age, sex, race/ethnicity, education, smoking, drinking, occupational and off-work noise exposure.
